# The serine protease HtrA regulates Group B *Streptococcus* virulence and affects the host response to infection

**DOI:** 10.1371/journal.ppat.1013562

**Published:** 2025-10-06

**Authors:** Alyssa Brokaw, Grace Wallen, Austyn Orvis, Hei Joon Kwon, Ravin Seepersaud, Shayla Nguyen, Kavita Sharma, Michelle Coleman, Phoenicia Quach, Joy Twentyman, Jay Vornhagen, Lisa A. Jones, Chenwei Lin, Philip R. Gafken, Lakshmi Rajagopal

**Affiliations:** 1 Center for Global Infectious Disease Research, Seattle Children’s Research Institute, Seattle, Washington, United States of America; 2 Department of Global Health, University of Washington, Seattle, Washington, United States of America; 3 Proteomics & Metabolomics Shared Resource, Fred Hutchinson Cancer Center, Seattle, Washington, United States of America; 4 Department of Pediatrics, University of Washington, Seattle, Washington, United States of America; Boston Children's Hospital, UNITED STATES OF AMERICA

## Abstract

Group B Streptococcus (GBS) rectovaginally colonizes up to 20% of women worldwide and is a leading cause of invasive infections during pregnancy, contributing annually to a significant proportion of preterm births, neonatal infections, and stillbirths. Despite its reputation as a perinatal pathogen, GBS infection rates in non-pregnant adults are also increasing. While much progress has been made to understand transcriptional regulation of virulence by two-component systems, many aspects of GBS virulence regulation remain understudied. Although many bacterial pathogens utilize high temperature response A (HtrA) family serine proteases to regulate virulence and stress responses through varied mechanisms, the function of HtrA in GBS was unknown. Here, we demonstrate that HtrA is localized to the GBS membrane and regulates the abundance of endogenous surface and secreted proteins, including a subset of virulence factors. Although deletion of *htrA* (Δ*htrA*) increased dissemination to placentas and fetuses, this strain caused significantly fewer adverse pregnancy outcomes compared to isogenic wild-type (WT). Placentas from Δ*htrA*-infected dams contained more chemokines, pro-inflammatory IL-1β, and neutrophil myeloperoxidase than isogenic WT-infected placentas, suggesting that Δ*htrA* GBS induces potent neutrophil chemotaxis. However, immunosuppressive IL-10 was present at increased concentration, which may in part explain the attenuation of adverse pregnancy outcomes during Δ*htrA* infection. Finally, we note that recombinant GBS HtrA directly cleaves human fibronectin *in vitro*, highlighting that this protease may also target host substrates during infection. Together, these findings support a role for HtrA as a post-translational regulator of GBS virulence and suggest that inhibiting HtrA activity may hold therapeutic promise against GBS induced adverse pregnancy outcomes.

## Author summary

During pregnancy, Group B *Streptococcus* (GBS) infections cause preterm birth, neonatal infection, and stillbirth. Thus, therapeutics to prevent GBS infections are needed. Here we characterized the impact of the bacterial protease HtrA on GBS infection using mouse models that mimic *in utero* transmission during pregnancy. Despite high burden in fetal tissues, GBS lacking HtrA (Δ*htrA*) caused fewer adverse pregnancy outcomes. In fact, placentas from Δ*htrA*-infected mice contain more immunosuppressive IL-10. This suggests that HtrA may control inflammatory pathways that promote preterm birth during GBS infection. This overall reduction in pathogenicity supports HtrA’s role regulating GBS surface proteins, as multiple virulence factors displayed altered abundance in Δ*htrA* GBS. Additionally, we show that GBS HtrA can directly process the extracellular matrix protein fibronectin, an abundant placental substrate whose processing by HtrA could both restrict GBS binding to placental cells and modulate the placental immune response. Together, our data supports a model in which HtrA shapes the GBS proteome – and likely the host immune response – to enhance pathogenesis. These findings could inform the development of inhibitors targeting HtrA, which could reduce adverse effects of GBS infections.

## Introduction

Approximately 20% of women are rectovaginally colonized by Group B Streptococcus (GBS, *Streptococcus agalactiae*). Despite being an asymptomatic colonizer, GBS can be a major health threat during pregnancy. Approximately 15% of neonates born each year are exposed to maternal GBS [1], and *in utero* GBS infections are estimated to cause up to 1 million cases of preterm birth (PTB) and 50,000 stillbirths annually [2,3]. Invasive GBS infections cause up to 400,000 cases of pneumonia, sepsis, and meningitis in neonates and infants each year [1,3]. Beyond its major impact on maternal and infant health, GBS invasive disease rates are also rising in non-pregnant adults [4].

Treatment of GBS infections is currently restricted to antibiotics. Accordingly, some countries administer intrapartum antibiotic prophylaxis during delivery to reduce rates of vertical transmission [5]. While recent research has focused on the development of a GBS vaccine [5], it is clear that alternative strategies for GBS prophylaxis and treatment are needed amid rising rates of antibiotic resistance among clinical isolates [6].

Anti-infection strategies typically target pathways essential for pathogen survival or instead target virulence factors. GBS encodes a large arsenal of virulence factors whose impact on disease varies by site of infection, GBS strain, and sequence type [5,6]. Additionally, GBS encodes many two-component systems that regulate gene transcription in response to environmental stimuli, facilitating bacterial adaptation to its new niche [7]. Despite our current understanding of these specific pathways in GBS, transcriptional control of virulence can occur through multiple overlapping mechanisms that are difficult to disentangle. While the major GBS virulence regulator known as CovR/S is well described [7], major knowledge gaps remain surrounding the roles of other virulence regulating mechanisms including those that occur post-transcriptionally. Dissecting the roles of such pathways could pave the way for the development of alternative therapeutic strategies to reduce GBS burden.

The high temperature response A (HtrA) family of serine proteases is broadly conserved among bacterial pathogens. These proteins contain a trypsin-like protease domain with a histidine-aspartic acid-serine (H-D-S) catalytic triad that is essential for enzyme activity, along with one to two C-terminal PDZ domains that are involved in substrate recognition [8–10]. HtrA was first described in *Escherichia coli*, which encodes three homologs (DegP, DegQ, and DegS) that recognize and degrade misfolded proteins to regulate bacterial virulence and stress responses [11–13]. More recently, similar roles have been reported for HtrA homologs in other bacteria. Deletion of *htrA* is frequently associated with attenuated virulence and these strains often exhibit heightened susceptibility to environmental changes such oxidative, pH, or high temperature stresses [8–10,14–20]. Despite the established role of HtrA in bacterial virulence and stress, the specific mechanisms through which HtrA contributes to these phenotypes remain understudied.

The mechanistic influence of HtrA on virulence and stress responses depends on the enzyme’s localization within the bacterial cell. Most Gram-negative bacteria secrete HtrA into the extracellular space [8,9]. Consequently, HtrA of *E. coli*, *Salmonella enterica*, *Shigella flexneri*, and *Helicobacter pylori* directly cleave host E-cadherin to weaken adherens junctions in the gut epithelium and permit bacterial translocation. Cleavage of other host extracellular matrix (ECM) proteins in the gut lamina propria can further enhance dissemination [16,21]. In contrast, HtrA from Gram-positive bacteria are membrane-anchored. As such, homologs of *Listeria monocytogenes, Bacillus anthracis, Bacillus subtilis*, *Streptococcus pneumoniae*, *Streptococcus mutans*, and *Streptococcus pyogenes* largely target endogenous proteins at proximity to the bacterial cell surface, with Gram-positive HtrAs being frequently linked to the quality control of exported virulence factors such as adhesins, peptide pheromones, and even other proteases [14,22–27]. Despite extensive literature on the impact of HtrA on physiology and pathogenesis of some pathogens, including assessment in multiple pathogenic Streptococci, how HtrA influences GBS virulence and the accompanying host response remains unknown.

In this study, we show that GBS HtrA is localized to the cell membrane and regulates the abundance of endogenous membrane-localized and secreted proteins including multiple virulence factors. We validated that one such protein known as Streptococcal surface immunogenic protein (Sip) is directly processed through the proteolytic activity of HtrA. In a mouse model of pregnancy-associated ascending infection, an *htrA* deletion mutant (Δ*htrA*) caused fewer adverse pregnancy outcomes despite increased bacterial replication in fetal tissues compared to isogenic WT infection. We propose that attenuation of adverse pregnancy outcomes occurs in part due to the high concentration of immunosuppressive IL-10 recovered from Δ*htrA* infected placentas. We also demonstrate that GBS HtrA can cleave human fibronectin (Fn) and speculate that HtrA-mediated Fn cleavage may regulate GBS dissemination and activate host inflammatory pathways associated with preterm labor (PTL). This study represents the first characterization of GBS HtrA as a post-translational regulator of GBS virulence. Additional detailed assessment of HtrA function and the identification of pathogenic processes reliant on its protease activity could inform the development of a small molecule inhibitor that could synergize with antibiotics and vaccines to prevent and treat a wide range of GBS clinical manifestations.

## Results

### HtrA is localized to the GBS membrane

**Fig 1:**
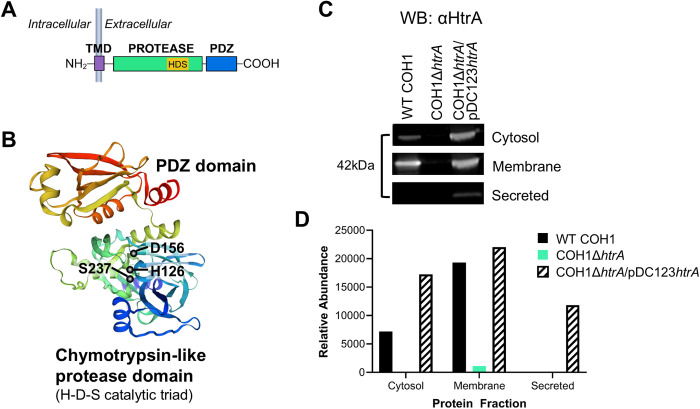
HtrA is primarily localized to the GBS membrane. (A) GBS HtrA contains an N-terminal putative transmembrane domain (TMD), protease domain with histidine-aspartic acid-serine (HDS) catalytic triad, and C-terminal PDZ domain (Postsynaptic density protein of 95 kDa; *Drosophila* disc large tumor suppressor; Zonula occludens-1 protein). Protein orientation was predicted by DeepTMHMM. (B) Predicted ribbon diagram of GBS HtrA with catalytic triad residues shown. (C) Western blot of cytosolic, membrane, and secreted protein fractions isolated from WT COH1, COH1Δ*htrA*, or COH1Δ*htrA/*pDC123-*htrA* GBS. HtrA was detected using rabbit serum raised against recombinant catalytically inactive GBS HtrA_S237A_. Blots were performed in biological triplicate and data shown is representative. (D) Densitometry of blot in panel C. The diagram in panel (A) was created with Biorender.com.

HtrA is a serine protease conserved among sequenced GBS strains, including the hypervirulent GBS isolate COH1 (serotype III, sequence type 17) used in this study. The domain organization of GBS HtrA is similar to that of other Gram-positive HtrA homologs, comprising an N-terminal signal peptide, a putative transmembrane domain, a chymotrypsin-like protease domain containing a conserved histidine-aspartic acid-serine (HDS) catalytic triad, and a C-terminal PDZ domain (Fig 1A). Hidden Markov modeling [28] predicted that GBS HtrA is localized to the membrane facing extracellularly. Structural modeling confirmed spatial clustering of the catalytic triad residues H126, D156, and S237 (Fig 1B). To identify the localization of GBS HtrA, WT COH1, an isogenic *htrA* deletion mutant (COH1Δ*htrA*), and the complemented mutant strain (COH1Δ*htrA*/pDC123*htrA*) were grown to mid-logarithmic phase and fractionated to collect cytosol, membrane, and secreted proteins. Western blot analysis identified a major reactive band of 42 kDa primarily within the membrane fraction of WT COH1 that was absent in COH1Δ*htrA*. HtrA expression was restored in the complemented strain (Fig 1C and 1D). Collectively, these findings confirm association of GBS HtrA with the bacterial membrane.

### HtrA mediates adverse outcomes during GBS pregnancy-associated infection

**Fig 2:**
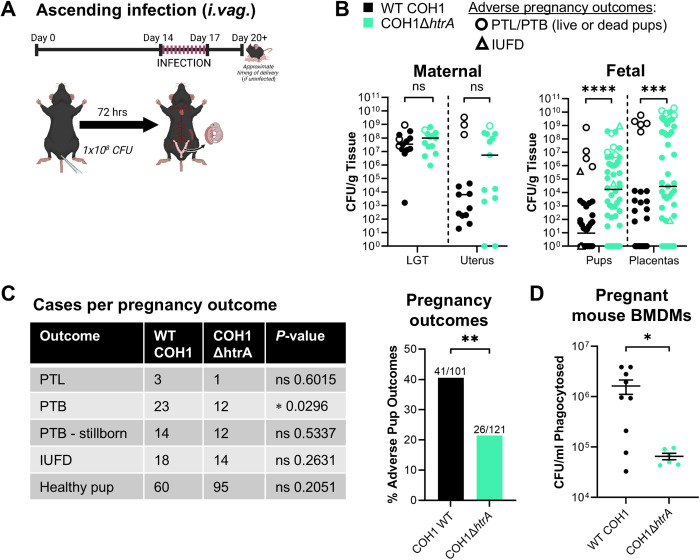
HtrA-deficient GBS is associated with fewer adverse pregnancy outcomes despite increased dissemination to fetal tissues. (A) Schematic of our murine model of pregnancy-associated ascending GBS infection. Pregnant C57BL/6J mice were infected intravaginally (*i.vag.*) with ~1x10^8^ CFU WT COH1 or COH1Δ*htrA* GBS (n = 13/strain) at gestational age E14. CFU were enumerated from maternal lower genital tract (LGT) and uterus and from fetal tissues (proximal and distal pups and placentas) at 72 hours post-infection or at preterm labor (PTL) onset. Pup viability was noted at necropsy. (B) CFU were normalized to tissue weight and medians are shown. Filled circles indicate healthy pregnancies and empty shapes indicate adverse outcomes. Significant differences were determined using Mann-Whitney test: ns *P* > 0.05, **** *P* < 0.0001. (C) Table shows cases of adverse pregnancy outcomes experienced by GBS-infected dams (PTL only) and their pups. Graph indicates percentage of pups experiencing any adverse outcome (PTB, stillbirth, or intrauterine fetal death (IUFD)) out of the total pups per group. Contingency tables were used to compare healthy versus adverse case numbers and significance was assessed via Fisher’s exact test: ns *P* > 0.05, * *P* < 0.05, ** *P* < 0.01. (D) BMDMs were differentiated *in vitro* following the isolation of bone marrow stem cells from pregnant C57BL/6J mice at gestational age E18. BMDMs generated from each dam were infected with WT COH1 versus COH1Δ*htrA* GBS at MOI 5 for 30 minutes prior to treatment with penicillin G and gentamycin. Cells were washed in PBS, lysed using 0.1% (v/v) Triton-X, and dilution plated to enumerate phagocytosed bacteria. Each datapoint represents a single GBS biological replicate with data from all 3 pregnant dams included. Means ± SEM were assessed for significance using a two-tailed unpaired student’s t-test: * *P* < 0.05. The diagram in panel (A) was created with Biorender.com.

Given that GBS is an important perinatal pathogen, we next sought to determine how *htrA* deletion impacts virulence during pregnancy using a murine model of ascending infection as described previously [29]. After first confirming that COH1Δ*htrA* exhibited no *in vitro* growth defects compared to WT (S1 Fig), we intravaginally infected pregnant dams with ~1x10^8^ CFU of WT COH1 or COH1Δ*htrA* at embryonic day 14 (E14) approximating the late second to early third trimester. Mice were sacrificed at the onset of preterm labor (PTL) or at 3 days post-infection. At endpoint, maternal (lower genital tract (LGT) and uterus) and fetal tissues (proximal and distal pups of the left and right uterine horn plus respective placentas) were collected to evaluate GBS vertical transmission (Fig 2A). Occurrence of adverse pregnancy outcomes was also noted, including cases of PTL (experienced by dam) or PTB (experienced by pup) (defined as vaginal bleeding or pups in the cage), stillbirths (dead pups in the cage following PTB), and intrauterine fetal deaths (IUFD, including both intact unresponsive and resorbing fetuses).

While deletion of other bacterial *htrA* homologs is typically associated with an attenuation of virulence [9], we observed no significant differences in LGT colonization or ascension to the uterus (Fig 2B, Maternal). However, we were surprised to see that GBS burden was significantly elevated in fetal tissues from dams infected with COH1Δ*htrA* compared to the isogenic COH1 WT (Fig 2B, Fetal). Two litters in the WT COH1 infection group were stillborn during the dark cycle and we were unable to enumerate their GBS burden due to infanticide. The single preterm litter in the COH1Δ*htrA* group was stillborn, but delivery during the light cycle permitted analysis of GBS burden in these tissues. Thus, the overall burden is likely underestimated during WT COH1 infection.

When accounting for the total number of pups per group, the rate of adverse pup outcomes from dams infected by COH1Δ*htrA* was nearly half of that observed from WT COH1 dams (Fig 2C, graph). This result was primarily driven by a significantly higher rate of PTB during COH1Δ*htrA* infection (Fig 2C), but improved survival of litters likely also contributed. We observed no significant differences when comparing rates of PTL, stillbirth, or IUFD between infection groups. Although COH1Δ*htrA* GBS was not attenuated for replication in fetal tissues, this strain was significantly attenuated in its ability to cause adverse outcomes. These data show that although HtrA is dispensable for GBS vertical transmission and dissemination *in vivo*, its expression is correlated with PTB.

### BMDMs from pregnant mice insufficiently phagocytose HtrA-deficient GBS

The importance of phagocytes in host defense against GBS infections is well documented [29–35]. To examine if macrophages respond differently to WT COH1 versus COH1Δ*htrA* GBS during infection, we utilized bone marrow-derived macrophages (BMDMs). These cells were differentiated from stem cells collected at embryonic day E18 from naïve pregnant mice. Following *in vitro* differentiation, we observed that these BMDMs more readily phagocytosed WT COH1 compared to COH1Δ*htrA* (Fig 2D). Combined with the *in vivo* data, these findings suggest that while phagocytes at the maternal-fetal interface may more effectively restrain WT COH1 GBS, the tradeoff of this interaction may be the activation of inflammatory pathways that are detrimental to maintaining a term pregnancy.

### HtrA deletion changes the murine placental immune response against GBS

Previous studies show that a pro-inflammatory immune response in the maternal-fetal interface is strongly associated with adverse pregnancy outcomes during GBS infection [30,31,36–38]. We hypothesized that the high rate of adverse pregnancy outcomes observed during WT COH1 infection could be explained by elevated pro-inflammatory responses during COH1Δ*htrA* infection. Thus, we quantified chemotactic, pro-inflammatory, and anti-inflammatory factors in the placental lysates.

**Fig 3:**
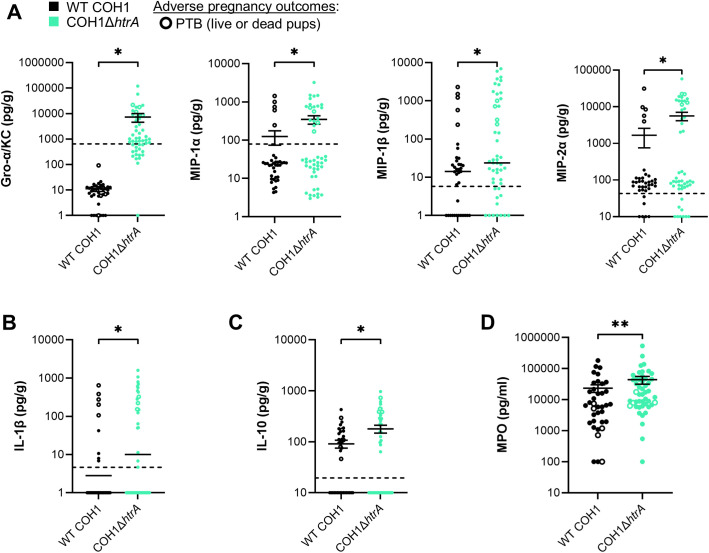
HtrA deletion changes the murine placental immune response to GBS. (A–C) Mouse placental lysates were assessed by Luminex to quantify (A) chemotactic, (B) pro-inflammatory, and (C) immunosuppressive factors. All data was normalized to tissue weight and each point represents a single placenta with empty circles indicating cases of preterm birth (PTB) . The dashed line shows mean analyte levels from PBS-treated control mice. Graphs represent means ± SEM with significant differences determined by two-tailed unpaired student’s t-tests: * *P* < 0.05. Trending statistics (*P* < 0.1) are also indicated. (D) neutrophil myeloperoxidase (MPO) was quantified from mouse placental lysates by ELISA. Graph depicts means ± SEM with significance determined using two-tailed unpaired student’s t-test: ** *P* < 0.01.

We observed significantly elevated Gro-α/KC, MIP-1α, MIP1-β, and MIP-2α chemokines in placental lysates from dams infected with COH1Δ*htrA* compared to WT COH1 (Fig 3A). In particular, Gro-α/KC levels were 1,000-fold higher during COH1Δ*htrA* placental infection. The pro-inflammatory cytokine IL-1β was also significantly more concentrated in placentas from COH1Δ*htrA* infections versus WT COH1 infections (Fig 3B). While these immunomodulators typically facilitate immune activation associated with bacterial clearance, we also detected a significant and concomitant increase in anti-inflammatory IL-10 in COH1Δ*htrA* infected placentas relative to WT COH1 (Fig 3C). We predict that this suppressive IL-10 signal may both hinder phagocyte-mediated clearance and contribute towards the low rates of adverse pregnancy outcomes observed during infection with COH1Δ*htrA*.

Among phagocytes, neutrophils are paramount for early host defense against GBS perinatal infection [30–35]. Due to a 1,000-fold increase in neutrophil chemotactic factor Gro-α/KC [39] in placentas from COH1Δ*htrA* infections, we examined concentrations of the neutrophil-specific enzyme myeloperoxidase (MPO) as a proxy for neutrophil recruitment. As expected, we detected significantly more MPO in placentas from COH1Δ*htrA* infection compared to those from WT COH1 (Fig 3D), suggesting that the heightened Gro-α/KC signal is functional and facilitates neutrophil chemotaxis to the COH1Δ*htrA*-infected placenta. Despite their presence, these neutrophils appear to be attenuated in their ability to clear GBS during COH1Δ*htrA* infection, leading to the significantly increased burdens we observed in these tissues.

### HtrA alters the GBS surface proteome

**Fig 4:**
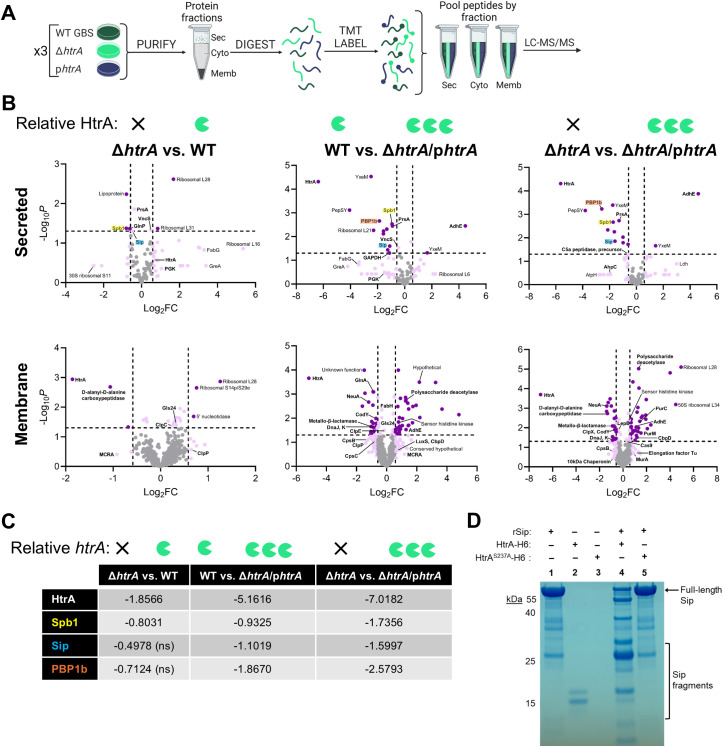
HtrA affects global GBS protein abundance. (A) Schematic of proteomic study comparing abundance of proteins from cytosolic, membrane, and secreted fractions isolated from three biological replicates of WT COH1, COH1Δ*htrA*, and COH1Δ*htrA* pDC123/*htrA* GBS grown in TSB. Tandem mass tag (TMT) labeling was used to multiplex samples by protein fraction prior to liquid chromatography-mass spectrometry (LC-MS/MS). (B) Matrix of volcano plots showing pairwise GBS strain comparisons analyzed for the indicated protein fraction. Characters above each graph indicate relative HtrA levels of each strain. Proteins with significant changes in abundance (fold-change (FC) ≥ 1.5 & *p-*value < 0.05) are shown in dark purple and proteins meeting only one significance criterion are lilac. Bolded proteins have been previously linked to bacterial virulence and/or stress responses. (C) Table summarizes HtrA-mediated dose-dependent changes in abundance for selected proteins (highlighted in the corresponding color in the above volcano plots). Values noted ns were not significant for the indicated strain comparison. (D) Cleavage assay assessing interaction of recombinant HtrA-H6 and catalytically inactive HtrA^S237A^-H6 with the putative endogenous substrate Sip (1:1000, HtrA:Sip). Coomassie stained gel indicates full-length Sip and fragments generated by cleavage compared to single protein controls (lanes 1-3). The experiment was repeated three times and a representative image is shown. The schematic in panel (A) was created with Biorender.com.

Due to previous reports that HtrA homologs from Gram-positive pathogens regulate endogenous bacterial proteins [22–27,40], we examined if GBS HtrA influences the abundance and distribution of proteins within its proteome. To this end, we employed quantitative mass spectrometry-based proteomics using tandem mass tag (TMT)-labeling to profile secreted, membrane, and cytosolic proteins fractionated from WT COH1 (expressing normal levels of HtrA), COH1Δ*htrA* (devoid of HtrA), and COH1Δ*htrA*/pDC123*htrA* (overexpressing HtrA) (Fig 4A).

In total, approximately 1,000 GBS proteins were detected by mass spectrometry, accounting for approximately half of the COH1 proteome (~2,003 ORFs in the COH1 genome) (S1 Data). Of those, 110 exhibited significant changes in abundance due to loss or over-expression of *htrA* (Figs 4B and S2, also see S2 Data). Fold-change and *p*-values for all proteins can be found in S2 Data. Of note, the greatest fold-change (FC) was seen for HtrA itself, confirming the integrity of our *htrA* deletion and over-expression strains. We hypothesized that some proteomic changes could be due to HtrA directly and that the changes in abundance of these putative substrates would be of greater magnitude when comparing strains with a larger relative difference in *htrA* expression levels. Consequently, the following strain comparisons were made to better identify GBS HtrA targets: COH1Δ*htrA* vs. COH1Δ*htrA*/pDC123*htrA* (representing the largest difference in *htrA* expression), COH1 WT vs. COH1Δ*htrA*/pDC123*htrA*, and COH1Δ*htrA* vs. COH1 WT (representing the smallest difference). Consistent with this hypothesis, a greater number of hits were obtained when comparisons included larger relative differences in *htrA* expression between the strains (S2C Fig). Notably, reduced abundances of secreted pilus island 2-b backbone protein (Spb1), Streptococcal surface immunogenic protein (Sip), and penicillin-binding protein 1b (PBP1b) were observed in a dose-dependent manner relative to *htrA* expression (Fig 4B and 4C), suggesting that these proteins may be direct substrates of HtrA.

Among the proteins that underwent the largest FC were aldehyde-alcohol dehydrogenase (AdhE, Log_2_FC 4.6, secreted), a hypothetical protein with PepSY domain (GBSCOH1_0641, -4, secreted), and multiple ribosomal proteins (Fig 4B). Interestingly, 77% of our observed significant changes occurred within the membrane and secreted fractions (S2C Fig), supporting a predominant role for HtrA in the processing of the GBS surface proteome. Interestingly, most of the secreted proteins were reduced in abundance in the absence of HtrA (17/21, 81%), while most membrane proteins increased in abundance (44/73, 60%).

### Abundance of virulence-associated proteins is regulated by GBS HtrA

To identify bacterial pathways that may be enriched in our proteomics dataset due to HtrA-mediated changes in protein abundance, we performed KEGG analysis. Based on this assessment, we found HtrA to be important for the regulation of a variety of GBS processes. The top four KEGG functional categories of HtrA regulated proteins included ribosome (n = 16), ABC transport (n = 9), glycolysis/gluconeogenesis (n = 5), and purine or pyrimidine metabolism (n = 7) (S2 Data). While virulence is not a functional category in the KEGG database, 31 of our hits have either well-established roles in GBS pathogenesis or virulence associations in other bacterial pathogens. 15 of these virulence proteins displayed an increase in abundance in strains with lower *htrA* expression (Table 1) while 18 virulence proteins showed reduced levels (Table 2). The alterations in abundance of these virulence proteins may contribute to the increased resistance to macrophage phagocytosis and altered pregnancy-associated virulence phenotypes observed for COH1Δ*htrA* GBS.

**Table 1:** A selection of virulence-associated proteins with increased abundance in strains with low vs. high relative *htrA* expression.

Gene Locus	Gene product/BLAST result	Proposed pathogenesis role	Organism (ref)
GBSCOH1_2002	Serine protease, HtrA	Virulence-associated protease	Various [8,9] GBS (this study)
GBSCOH1_0053	Aldehyde-alcohol dehydrogenase, AdhE	Ethanol tolerance Adhesin*	*S. pneumo* [41] *S. suis* [42]
GBSCOH1_0364	Copper-translocating P-type ATPase, CopA	Increased tolerance to copper intoxication	GBS [43]
GBSCOH1_0373	Sensor histidine kinase, BgrS or BaeS	Reduced β antigen expression Cell envelope stress	GBS [44] *E. coli* [45]
GBSCOH1_0747	Phosphoenolpyruvate-protein phosphotransferase, PtsA	Adhesin* Virulence gene regulation	*S. pneumo* [46,47]
GBSCOH1_0023	Phosphoribosylaminoimidaole-succinocarboxamide synthase, PurC	Unknown – disruption in purine biosynthesis attenuates survival	*S. pneumo* [48] *E. coli* [49]
GBSCOH1_1211	Choline binding protein D, CbpD	Competence & fratricide	*S. pneumo* [50]
GBSCOH1_2003	Partitioning protein, ParB family	SOS response Gene regulation	*Pseudomonas* [51]
GBSCOH1_0963	Cardiolipin synthetase, Cls	Promotes lipid remodeling Antibiotic resistance	*S. mutans* [52] *S. aureus* [53]
GBSCOH1_1343	SsrA-binding protein, SmpB	Unknown – disruption attenuates intracellular survival	*Listeria* [54]
GBSCOH1_1580	Universal stress protein family, Usp	Adhesin* Stress tolerance	*S. aureus* [55,56]
GBSCOH1_1612	Phosphoglycerate kinase, PGK	Adhesin *	GBS [57,58]
GBSCOH1_0964	Formate-tetrahydrofolate ligase, Fhs	Unknown – necessary for colonization & dissemination	*S. suis* [59]
GBSCOH1_0329^#^	3-oxoacyl-(acyl-carrier-protein) synthase II, FabF	Fatty acid synthesis	*S. aureus* [60]
GBSCOH1_0715^#^	Superoxide dismutase, SodA	Oxidative stress Intracellular survival	GBS [61] *S. suis* [62]

All proteins are significant by FC ≥ 1.5 and *P* < 0.05) cutoffs.

Proteins were categorized as virulence-associated through literature searches.

*Cytosolic protein with published moonlighting functions at the bacterial surface.

# Protein had increased abundance in COH1Δ*htrA* cytosol vs. COH1 WT, but decreased abundance in COH1 WT vs. COH1Δ*htrA*/pDC123*htrA* cytosol.

**Table 2:** A selection of virulence-associated proteins with reduced abundance in strains with low vs. high relative *htrA* expression.

Gene Locus	Gene product/BLAST result	Proposed pathogenesis role	Organism (ref)
GBSCOH1_0161	Penicillin-binding protein 1b, putative, PBP1b	Cell wall remodeling Antimicrobial peptide resistance	Various [63]
GBSCOH1_1614	Glyceraldehyde-3-phosphate dehydrogenase, GAPDH	Immunomodulatory protein/Adhesin*	GBS [64–67]
GBSCOH1_0031	Streptococcal surface immunogenic protein, Sip	Immunogenic protective antigen	GBS [68–70]
GBSCOH1_1279	Pilus island-2b backbone protein, Spb1	Colonization factor Adhesion/invasion Resistance to phagocytosis	GBS [71,72]
GBSCOH1_0734	Rotamase family protein, PrsA	Chaperone/folding of virulence factors	GAS [73] *Listeria* [15]
GBSCOH1_0094	D-alanyl-D-alanine carboxypeptidase family protein	Peptidoglycan turnover Stress tolerance	*S. pneumo* [74] *Francisella* [75]
GBSCOH1_1064	CMP-N-acetylneuraminic acid synthetase, NeuA	Capsule biosynthesis Complement evasion	GBS [76,77]
GBSCOH1_0099	Chaperone protein, DnaJ	Immunomodulatory protein*	*S. pneumo* [78]
GBSCOH1_0098	Chaperone protein, DnaK	Stress tolerance Adhesin*	*S. mutans* [79] *Neisseria* [80]
GBSCOH1_1675	ATP-dependent Clp protease, ATP-binding subunit, ClpP	Modulator of virulence gene expression	*S. pneumo* [81,82]
GBSCOH1_1609	Glutamine synthetase, type I, GlnA	Colonization factor Adhesin*	*S. suis* [83]
GBSCOH1_1526	Transcriptional regulator, CodY	Regulates virulence gene expression	GBS [84]
GBSCOH1_0049	Holliday junction DNA helicase, RuvB	Alters virulence gene expression Promotes intracellular survival	*S. enterica* [85]
GBSCOH1_1290	Nucleotidyl transferase, putative, IspD2	Adhesin*	*S. aureus* [55]
GBSCOH1_1607	Metallo-beta-lactamase superfamily protein, Rnj	Regulates virulence gene expression	*E. faecalis* [86]
GBSCOH1_0688	Translation elongation factor, Tu	Adhesin*	*S. aureus* [87]
GBSCOH1_0329^#^	3-oxoacyl-(acyl-carrier-protein) synthase II, FabF	Fatty acid synthesis	*S. aureus* [60]
GBSCOH1_0715^#^	Superoxide dismutase, SodA	Oxidative stress Intracellular survival	GBS [61] *S. suis* [62]

All proteins are significant by FC ≥ 1.5 and *P* < 0.05) cutoffs.

Proteins were categorized as virulence-associated through literature searches.

*Cytosolic protein with published moonlighting functions at the bacterial surface.

# Protein had increased abundance in COH1Δ*htrA* cytosol vs. COH1 WT, but decreased abundance in COH1 WT vs. COH1Δ*htrA*/pDC123*htrA* cytosol.

### Sip is an endogenous substrate of GBS HtrA

We next sought to validate our proteomic findings by determining whether proteins shown to be altered with varied *htrA* expression are direct substrates of this protease. To this end, we purified recombinant WT (HtrA-H6) and a proteolytically inactive variant (HtrA^S237A^-H6) HtrA (S3A Fig). After validating the activity of these recombinant proteins against the model protease substrate β-casein (S3B and S3C Fig), we interrogated Sip as a possible HtrA substrate due to its dose-dependent abundance in our proteomic screen. Co-incubation of Sip with HtrA-H6 resulted in the generation of fragments ranging from ~25 to <10 kDa. No processing was visible following co-incubation with HtrA^S237A^-H6. Collectively, these findings validate a hit from our proteomic experiment and demonstrate that Sip is an endogenous substrate of the GBS serine protease HtrA.

### GBS HtrA cleaves human fibronectin

**Fig 5:**
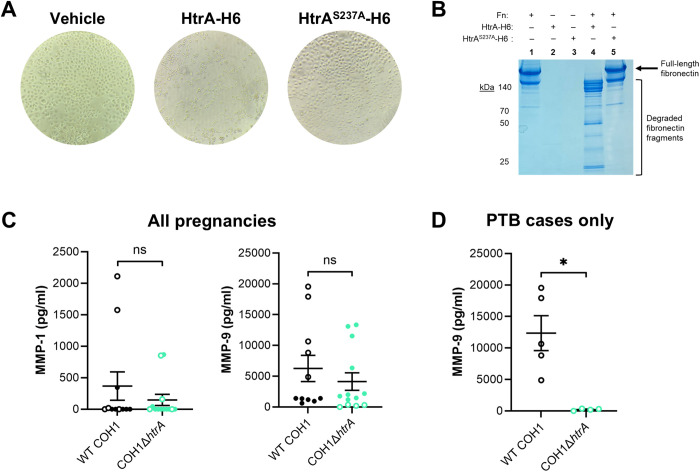
HtrA activity against host Fn may promote matrix metalloproteinase-mediated PTL. (A) Brightfield micrographs of human vaginal epithelial cells treated with vehicle control, recombinant HtrA-H6, or HtrA^S237A^-H6. (B) Cleavage assay assessing interaction of HtrA-H6 and catalytically-inactive HtrA^S237A^-H6 with the putative host substrate fibronectin (Fn) (1:1000, HtrA:Fn). Coomassie stained gel indicates full-length Fn and fragments generated by cleavage compared to single protein controls (lanes 1-3). (C-D) Matrix metalloproteinase (MMP)-1 and MMP-9 quantified from mouse placental lysates by ELISA. Graphs represent means ± SEM with each point indicating a single placenta. Empty circles represent placentas from cases of PTB. Significant differences were determined using two-tailed unpaired student’s t-tests: ns *P* > 0.05, ** *P* < 0.01. Trending statistics (*P* < 0.1) are also indicated.

To examine whether GBS HtrA may also target host substrates during GBS infection, we treated human vaginal epithelial cells with HtrA-H6 or HtrA^S237A^-H6. HtrA-H6 but not the catalytically inactive form caused a “trypsinized” phenotype (Fig 5A), suggesting that GBS HtrA targeted host proteins involved in cell adhesion. We hypothesized that degradation of host extracellular matrix (ECM) components by GBS HtrA could result in tissue damage associated with the development of adverse pregnancy outcomes. Fibronectin (Fn) is an important constituent of the placental ECM and alterations to placental Fn structure are correlated with PTL and other adverse pregnancy outcomes [88]. Using our *in vitro* cleavage assay, we show that HtrA-H6 degraded full-length Fn (Fig 5B, lane 4). In contrast, no cleavage was observed following treatment with HtrA^S237A^-H6 (Fig 5B, lane 5), confirming Fn as a specific GBS HtrA host substrate.

### HtrA activity against host Fn may promote matrix metalloproteinase-mediated PTL

One effect of Fn cleavage at the maternal-fetal interface is the induction of PTL through COX-2-mediated eicosanoid production and release and activation of matrix metalloproteinases (MMP)-1 and -9 [89]. Our placental lysates were not flash frozen, so we could not quantify short-lived lipid eicosanoids in these samples. However, we could assess MMP-1 and MMP-9 concentrations by ELISA. Despite detecting no significant differences for either MMP between WT COH1 and COH1Δ*htrA* infected placentas (Fig 5C), we noted that placentas from cases of PTL skewed the MMP-9 results. Thus, we performed an additional analysis using only preterm placentas in each group. Interestingly, placentas from COH1Δ*htrA*-infected dams who experienced PTL exhibited ~ 40-fold less MMP-9 than those from isogenic WT-infected dams who also experienced PTL (Fig 5D). These findings suggest that while COH1Δ*htrA* appears to be capable of more rarely inducing PTL, the MMP-9 pathway may be activated during GBS infection in an HtrA-dependent manner.

**Fig 6:**
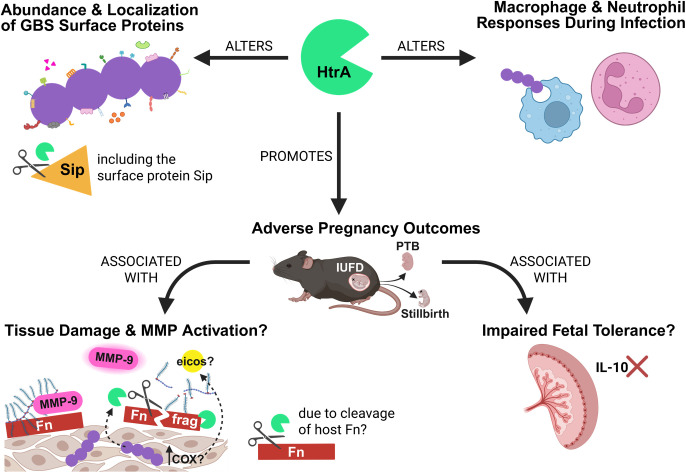
Model of the effect of HtrA on GBS perinatal virulence. HtrA regulates GBS virulence by altering both endogenous GBS and host proteins. HtrA mediated changes to the GBS surface proteome, including a subset of virulence factors. These findings support the partial attenuation of adverse pregnancy outcomes including intrauterine fetal death (IUFD), preterm birth/labor (PTB/PTL), and stillbirth in pregnant mice infected by COH1Δ*htrA*. While these proteomic changes may occur directly or indirectly, we provide *in vitro* evidence that the immunogenic surface protein Sip is an endogenous substrate of HtrA. Importantly, HtrA-dependent changes to the GBS surface proteome also appear to impact interaction with the host. While the mechanisms leading to heightened cytokine and chemokine production during COH1Δ*htrA* placental infection remain unclear, the resulting immune environment seems to impair GBS detection by macrophages and neutrophils. We noted reduced phagocytic activity of BMDMs against COH1Δ*htrA*, which could contribute towards its high burden *in vivo*. This impaired phagocyte function likely occurs through IL-10-mediated suppression. Additionally, neutrophils recruited to the COH1ΔhtrA-infected placenta may also be functionally impaired by IL-10. We also identified human fibronectin (Fn) as a host substrate of GBS HtrA and speculate that proteolytic modification of Fn by GBS HtrA may favor downstream signaling that leads to PTL during WT COH1 infection. This signaling likely involves the eicosanoid-MMP axis, as supported by our observation that placentas from WT COH1 infected dams experiencing PTL display elevated levels of MMP-9 compared to those infected by COH1Δ*htrA*. Overall, our study identifies the serine protease HtrA as a novel regulator of GBS virulence. This figure was created with Biorender.com.

In summary, these findings underscore the importance of HtrA as a post-translational regulator of GBS perinatal virulence, which is likely due to its activity on both endogenous bacterial substrates and host ECM proteins (Fig 6).

## Discussion

The HtrA serine proteases represent a family of bacterial proteases that can have broad target specificity to both endogenous bacterial and host proteins. While activity has been linked to roles in virulence for many pathogenic bacteria [8,9], this study represents the first in-depth analysis of the effects of the GBS HtrA homolog on its endogenous proteome, virulence potential, and ensuing host immune response. We show that GBS HtrA is membrane-localized where it acts upon specific GBS surface proteins and host factors, subsequently influencing GBS virulence during pregnancy-associated infections.

We observed an attenuation in virulence of COH1Δ*htrA* in a mouse model of pregnancy-associated ascending infection. While this attenuation is consistent with findings for *htrA* mutants of other bacterial pathogens [8,9], the phenotypes associated with attenuated adverse outcomes during perinatal infection were unique. Although a loss of HtrA led to fewer adverse pregnancy outcomes, we observed significantly higher bacterial burdens in fetal tissues isolated from dams infected by COH1Δ*htrA* compared to WT COH1. This attenuation in virulence could be partly explained by our observation that *htrA* deletion altered the abundance of multiple virulence factors as revealed by mass spectrometry profiling. Among these were previously characterized adhesins [65,66,71,72]. The altered abundance of these adhesins in COH1Δ*htrA* GBS suggests that despite its increased placental burden, this mutant strain may undergo fewer direct interactions with the host tissue compared to WT COH1.

We also observed large differences in the host response following ascending infection of COH1Δ*htrA* versus WT COH1 in pregnant mice. Specifically, placental immune responses exhibited increase levels of numerous signaling molecules including chemokines (Gro-α/KC, MIP-1α, MIP-1β, and MIP-2) and the pro-inflammatory cytokine IL-1β, which are strongly associated with neutrophil recruitment and innate immune cell function [39]. Concordantly, levels of the neutrophil-specific enzyme MPO, a measure of neutrophil activation, were also elevated in COH1Δ*htrA*-infected placentas compared to WT COH1. Together, these results suggest that COH1Δ*htrA* GBS is better detected by host immune cells than WT COH1. It is possible that increased immune recognition occurs during COH1Δ*htrA* infection as a result of its elevated burden in fetal tissues. An alternative explanation could be that the surface proteome of COH1Δ*htrA* GBS displays more antigenic epitopes compared to that of WT COH1, thus resulting in increased immune recognition and improved neutrophil chemotaxis. Although the causal relationship between these phenotypes remains unclear, these results mirror findings during *Pseudomonas aeruginosa* virulence. The *P. aeruginosa htrA* homolog, *mucD*, is important during corneal infection due to a role in the suppression of Gro-α/KC, MIP-2, and IL-1β-mediated neutrophil function [90]. The similarity of the immune profile induced during infection by *htrA*-mutant *P. aeruginosa* and GBS suggests that some HtrA may in part influence bacterial virulence by modulating neutrophil responses through a shared mechanism that warrants further study.

While our observation of elevated neutrophil MPO suggests that neutrophils can infiltrate to the COH1Δ*htrA*-infected placenta, we simultaneously detected increased levels of immunosuppressive IL-10, potentially hindering neutrophil-mediated killing efficiency in these placentas. IL-10 has been previously shown to regulate phagocytic function in multiple settings, including infection during pregnancy [39,91]. In concordance with this, BMDMs isolated from pregnant mice displayed reduced phagocytic activity towards COH1Δ*htrA* versus WT COH1. It seems likely that IL-10 could suppress neutrophil and macrophage-mediated pro-inflammatory pathways that might otherwise promote PTL and other adverse pregnancy outcomes [29–31,36,38]. Further, suppression mediated by IL-10 could impair phagocytosis and other bactericidal mechanisms, facilitating heightened replication of COH1Δ*htrA* GBS in fetal tissues of our mice. Together, these results suggest that HtrA activity promotes a pro-inflammatory immune response that more effectively controls GBS infection of fetal tissues. However, this response comes at a cost to the host since the disruption of tolerance at the maternal-fetal interface can have negative consequences for the fetus by inducing pathways associated with PTB.

Our proteomic analysis identified 110 proteins whose abundance changed due to *htrA* deletion or over-expression. The overwhelming majority of these changes affected proteins localized to the GBS membrane or secreted into the culture supernatant, supporting a role for HtrA in shaping the content of the GBS surface proteome that is consistent with observations for other Streptococcal pathogens [23–25,92]. We identified a subset of proteins with significantly reduced abundance that have been previously linked to virulence, including the pilus island 2b backbone protein Spb1, the sialic acid capsule-modifying enzyme NeuA, and the transcriptional regulator CodY [72,76,84]. CodY is reported to regulate adhesins in both GBS and *S. suis* and a GBS *codY* deletion mutant exhibits heightened biofilm production [84,93]. CodY abundance was low in strains with reduced *htrA* expression and could thus contribute to impaired phagocytosis and subsequent high bacterial burdens observed during COH1Δ*htrA* infection.

Interestingly, we did not note any significant changes in abundance for other GBS proteases such as ScpB, CspA, or ClpP, suggesting that other proteases may be unable to compensate for the loss of HtrA in these conditions. However, whether other proteases can compensate for HtrA loss during exposure to stress remains unknown. One limitation is that our proteomic screen covered only ~50% of the GBS proteome; thus, our findings likely represent an underestimation of the number of GBS proteins affected by HtrA. Improved coverage of the proteome or use of more sensitive methods such as proximity labeling mass spectrometry could uncover additional HtrA interactors with improved clarity.

Beyond some well-described GBS virulence factors, we also noted changes in abundance of multiple moonlighting proteins, defined here as proteins capable of performing unique dual functions in a localization-dependent manner. For example, when localized to the bacterial surface the cytoplasmic chaperone DnaJ can elicit immunomodulatory effects beyond its canonical chaperone function [78,79]. We noted reduced abundance of DnaJ and DnaK in the membrane of COH1Δ*htrA* or WT COH1 compared to the *htrA* over-expressing strain. While this finding suggests that HtrA may directly affect DnaJ and DnaK abundance at the GBS membrane, their surface function in GBS remains unknown. Similarly, we observed altered abundance of the cytoplasmic glycolytic enzymes glyceraldehyde-3-phosphate dehydrogenase (GAPDH) and phosphoglycerate kinase (PGK) at the GBS membrane, both of which possess biologically distinct functions at the GBS surface. GAPDH is reported to be immunomodulatory, stimulating IL-10 production that hinders neutrophil recruitment [64]. Interestingly, GAPDH abundance was highest in the membrane of our HtrA overexpressing strain, suggesting that HtrA-dependent changes to other GBS surface proteins are likely involved in skewing the immunosuppressive response observed during COH1Δ*htrA* placental infection. Interestingly, *S. pyogenes htrA* deletion was shown to alter abundance of cell wall-localized DnaK, enolase, GAPDH, and elongation factor Tu [24], suggesting that a conserved subset of moonlighting proteins may be subject to regulation by HtrA in Streptococcal pathogens.

In an effort to validate our HtrA-dependent proteomic screen, we confirmed the surface protein Sip as a direct substrate of GBS HtrA, supporting the dose-dependent relationship we observed between Sip abundance and *htrA* expression. Due to Sip’s surface localization and decreased abundance in GBS strains with relatively low HtrA levels, future directions will examine whether HtrA regulates Sip abundance through its chaperone and/or protease activities and whether these activities support quality control, protein folding, processing, or export of Sip. Further, the loss of Sip – an immunomodulatory protein [69] – may contribute toward the altered immune profile observed during COH1Δ*htrA* infection.

In addition to shaping the endogenous GBS surface proteome, we identified human Fn as a host substrate of GBS HtrA. While the cell junction molecule, E-cadherin, is a well characterized host substrate of other bacterial HtrA homologs [9,16], to our knowledge this is the first study to identify a host ECM substrate of a Streptococcal HtrA. Due to its prevalence in the maternal-fetal interface and its contribution to tissue organization [94], the GBS-Fn interaction could simultaneously promote and hinder GBS virulence through distinct mechanisms. We speculate that cleavage of Fn by HtrA could limit the binding of adhesins that bind ECM, impairing host detection while facilitating dissemination. Our lab has previously identified similar mechanisms for the GBS Fn-binding proteins SfbA and ScpB, the second of which has additional protease activity. During infection by WT GBS, activated mast cells produce chymase that cleaves host Fn. This protease diminishes SfbA-mediated adherence in gestational tissues, improving pregnancy outcomes during GBS infection [95]. Conversely, in systemic infection models GBS is entrapped in fibrin clots through the activity of a mast cell transglutaminase that cross-links ScpB to its Fn substrate, instead facilitating pathogen clearance by phagocytes [96]. These studies support the idea that, while interaction with host ECM is important during colonization, this interaction can be exploited to prevent successful GBS dissemination. Thus, HtrA-mediated cleavage of host Fn could in part facilitate the reduced burden of WT COH1 in our ascending infection model.

Finally, in addition to its role as a ligand for pathogen adhesins, Fn can also interact with host signaling molecules in tissues [94]. For example, Fn fragments generated during pregnancy are associated with increased eicosanoid production and activation of MMP signaling cascadees upstream of PTL onset [89,97]. While we did not detect significant differences in MMP-1 or MMP-9 levels in the placentas when comparing tissues from all infected mice, we noted that COH1Δ*htrA* infected placentas from PTB cases had significantly less MMP-9 than those from WT COH1 cases. Though we were unable to assess eicosanoid production in these placentas, we hypothesize that the eicosanoid-MMP pathway is at least partially blunted in COH1Δ*htrA* infected pregnant mice due to a loss in HtrA-generated Fn fragments that would otherwise allow for full MMP activation and downstream signaling events associated with PTL.

In this study we have identified HtrA as a novel protease important in the post-translational regulation of GBS virulence factors during pregnancy-associated infections. Our findings show that HtrA can modulate the abundance of a variety of surface or exported GBS proteins including proteins with immunomodulatory properties and roles in pathogenesis, which may explain the attenuation of adverse pregnancy outcomes observed during COH1Δ*htrA* infection in our ascending infection model*.* We validated Sip as an endogenous GBS substrate that is cleaved by HtrA and propose Fn as a host substrate whose modification may influence the activation of pathways liked to PTL. HtrA inhibitors have been identified for *E. coli*, *H. pylori*, *C. trachomatis*, and *L. monocytogenes* homologs, suggesting that HtrA proteases can be tractable drug targets [16,98–100]. Future studies evaluating GBS HtrA tractability could inform the development of a small molecule inhibitor that could synergize with antibiotics and vaccines to further reduce the global burden of GBS.

## Materials and methods

### Ethics statements

All mouse experiments were approved by the Seattle Children’s Research Institute’s Institutional Animal Care and Use Committee (protocol #00036) and were performed in strict accordance with recommendations from the *Guide for the Care and Use of Laboratory Animals, 8*^*th*^ *edition.*

### Bacterial strains

The GBS clinical isolate COH1 is a serotype III, sequence type 17 clone originally isolated from an infant with bacteremia [101]. GBS was cultured using tryptic soy agar or broth (TSA/TSB, Difco Laboratories) at 37°C with 5% CO_2_. Overnight cultures were diluted 1:10 and grown to an optical density at 600 nm (OD_600_) of 0.3 for infections and *in vitro* assays, or 0.6 for protein fractionation.

When necessary, antibiotics (Sigma-Aldrich) were used at the following concentrations for *E. coli* (kanamycin 50 μg/mL, erythromycin 300 μg/mL, chloramphenicol 10 μg/mL, or ampicillin 100 μg/mL) or for GBS (kanamycin 1000 μg/mL, erythromycin 5 μg/mL, or chloramphenicol 5–10 μg/mL).

### Construction of htrA mutant strain

*E. coli* DH5α (New England Biolabs) was used for cloning unless otherwise stated. Primer names are listed in parentheses and their sequences are listed in S1 Table. COH1 genomic sequences were taken from the HG939456.1 assembly on NCBI.

The *htrA* deletion mutant COH1Δ*htrA* was generated via allelic exchange as previously described [37]. In brief, an allelic exchange cassette was constructed by cloning 1 kb chromosomal upstream and downstream regions flanking *htrA* (*htrA* upstream F/R; *htrA* downstream F/R) surrounding a kanamycin resistance cassette (*kanR* F/R) into the temperature-sensitive vector pHY304 via Gibson assembly (cloning kit, New England Biolabs). The resulting plasmid, pHY304-Δ*htrA*, was introduced into COH1 GBS by electroporation [102]. Selection for allelic exchange mutants was performed as previously described [103]. Replacement of *htrA* with the kanamycin resistance cassette was confirmed by PCR, followed by Plasmidsaurus whole genome sequencing that did not identify additional mutations of concern.

For *htrA* overexpression *in trans*, *htrA* was amplified from COH1 genomic DNA (*htrA* comp F/R) and cloned into pDC123 (pDC123 F/R) via Gibson assembly. The resulting vector pDC123-*htrA* was introduced into COH1Δ*htrA* GBS by electroporation, generating COH1Δ*htrA*/pDC123*htrA*.

### GBS growth curves

GBS overnight cultures were diluted 1:20 in TSB and incubated at 37°C with 5% CO_2_. At various hours post-inoculation, cultures were serially diluted, and plated to enumerate viable CFU/mL. For each experiment, strains were tested in biological triplicate.

### GBS Gram stains

GBS overnight cultures were spread onto sterile, pre-warmed glass slides. Once dried, smears were Gram stained according to the manufacturer’s instructions (Epredia). Images were acquired on a BZ-X800 microscope (Keyence) using 100x oil immersion.

### GBS protein fractionation

GBS cultures (60 mL at OD_600_ 0.6) were pelleted at 3,000 x *g* at 4°C for 10 minutes. For analysis of secreted proteins, culture supernatants were filtered using a Millex-GP syringe filter (0.22 μm, Millipore) and concentrated to ~1/15^th^ volume via an Amicon Ultra centrifugal filter device (10 kDa, Millipore) prior to storage at -20°C for subsequent Western blot analysis. For preparation of samples used in proteomics analysis, cOmplete protease inhibitor cocktail tablets (1 tablet/ 10 mL, Roche) were added to filtered supernatant prior to Amicon concentration and precipitated overnight in 10 mM sodium chloride and 4x volume of acetone at -20°C (Sigma-Aldrich). Precipitated proteins were pelleted via centrifugation at 16,000 x *g* at 4°C for 20 minutes and after evaporation of residual acetone, solubilized in Dulbecco’s PBS (Corning) and applied to a PD-10 desalting column (Cytivia). Secreted protein fractions were quantified (BCA Protein Assay Kit, Pierce), adjusted to a final concentration of 1 mg/mL, and stored at -80°C until use.

For isolation of GBS membrane and cytosol fractions, GBS pellets were washed in PBS (Corning) and resuspended in lysis buffer (20 mM Tris-HCl, 10 mM magnesium chloride, 500 U DNase, 50 ng/mL RNase A (all Sigma-Aldrich), cOmplete mini protease inhibitor (1 tablet/5 mL, Roche)). GBS was lysed using a PreCellys 24 bead beater (Bertin Technologies) with 30 second pulses at power level 6 and 5 minutes cooling on ice between steps. Insoluble material was pelleted at 5,000 x *g* at 4°C for 5 minutes and the supernatant was transferred to an OptiSeal ultracentrifuge tube (Beckman Coulter) and pelleted at 60,000 x *g* at 4°C for 45 minutes to separate cytosolic and membrane fractions. Supernatant containing cytosolic proteins was further concentrated using an Amicon Ultra centrifugal filter (10 kDa, Millipore) while the pelleted membrane proteins were resuspended in 20 mM Tris pH 7.5, 2 mM EDTA, 10% glycerol (all Sigma-Aldrich). Protein fractions were quantified by BCA (Pierce), adjusted to a final concentration of 1 mg/mL, and stored at -80 prior to use.

### Structural predictions of GBS HtrA

Subcellular localization and orientation of GBS HtrA (GenBank ID: CDN67468.1) was predicted using DeepTMHMM. Annotated ribbon diagram of GBS HtrA amino acid amino acid positions 50–360 was generated using Swiss-PdbViewer build model function (CC BY-SA 4.0 license).

### Western blot analysis of HtrA

Proteins were boiled in 1x LDS sample buffer (Invitrogen) prior to SDS-PAGE separation on 4–20% NuPAGE Bis-Tris gels using MOPS-SDS running buffer (Invitrogen) and transferred onto nitrocellulose membrane (Invitrogen). Membranes were blocked in Intercept PBS blocking buffer (LI-COR), followed by overnight incubation at 4°C with rabbit polyclonal anti-HtrA antiserum (Lampire) diluted 1:750 in Intercept antibody diluent (LI-COR). Membranes were washed twice in PBS/0.5% Tween-20, and probed with IRDye 680RD-conjugated goat anti-rabbit IgG (LI-COR) for 45 minutes. Blots were imaged on an Odyssey CLx imager and analyzed using Image Studio (LI-COR). Images are representative of one Western blot performed in biological triplicate. Densitometry analysis was performed in Fiji.

### Murine pregnancy-associated ascending infection model

6-8 week old C57BL/6J female mice (Jackson Laboratory) were paired 1:1 with isogenic male breeders for 48 hours. At approximately embryonic day 15 (E15), pregnant mice were infected intravaginally (*i.vag.*) with ~1x10^8^ CFU GBS using a gel-loading pipette tip (Rainin) and monitored for signs of preterm labor (vaginal bleeding or pups in cage). At preterm labor onset or 72 hours post-infection, dams were euthanized and maternal cardiac blood, lower genital tract, and uterus were collected. The proximal and distal pups and placentas in both uterine horns were also collected and pup viability was assessed. Solid organs were homogenized (Omni International) and samples were serially diluted and plated on TSA to enumerate CFU. When required, contaminants were screened via ChromAgar StrepB (ChromAgar) and GBS CFU were adjusted accordingly. GBS CFU were normalized according to tissue mass. Adverse outcomes were calculated out of the total number of pups born from all dams within the infection group. If evidence of infanticide was apparent following overnight delivery, the total number of pups born was assumed to be the average litter size for the corresponding infection group.

Tissue homogenates were incubated 1:1 overnight at 4°C in tissue lysis buffer (150 mM sodium chloride, 15 mM tris, 1 mM magnesium chloride, 1 mM calcium chloride, 1% (v/v) Triton-x containing cOmplete mini protease inhibitor tablets (1 tablet per 5 mL, Roche). Tissue debris was pelleted by centrifugation at 3,000 x *g* for 15 minutes and supernatants were stored at -80°C for later analysis.

### Generation of mouse BMDMs and phagocytosis assay

Bone marrow-derived macrophages (BMDMs) were generated from femur and tibia bone marrow of C57BL/6J mice as described [29] with the following modifications. Cells were isolated from pregnant dams at E18 and seeded on non-TC-treated sterile bio-assay dishes (Corning) in stimulation media containing RPMI-1640 (Gibco), 10% FBS (ThermoFisher), 1x PenStrep (ThermoFisher), and 10 ng/mL recombinant mouse M-CSF (Miltenyi). On day 3, additional stimulation media was added and on day 7 cells were harvested and seeded into 48-well plates (Corning) at 2.5x10^5^ cells/mL.

The following day, the BMDMs were infected with GBS at MOI 5 and centrifuged for 5 minutes at 800 x *g* to synchronize the infection. At 30 minutes post-infection, 5 µg/mL penicillin G (Sigma-Aldrich) and 100 µg/mL gentamycin (Gibco) were added for 30 minutes to kill extracellular GBS. Finally, macrophages were washed, trypsinized, and lysed with 0.1% (v/v) Triton-x (Sigma-Aldrich). Lysates were serially diluted and plated to enumerate viable GBS CFU/mL. BMDMs were isolated from 3 pregnant dams and each was tested with separate GBS biological replicates in technical triplicate.

### Luminex analysis of murine tissue lysates

Tissue lysates from murine infections were thawed and centrifuged at 11,000 x *g* for 15 minutes to remove tissue debris. Luminex assays were performed according to manufacturer’s instructions (Mouse ProcartaPlex Multiplex immunoassay, ThermoFisher), using 25 μL of clarified tissue lysates to quantify the levels of 11 immuno-analytes (Gro-α/KC, IFN-γ, IL-1β, IL-10, IL-12p70, IL-6, MCP-1, MIP-1α, MIP-1β, MIP-2α, and TNF-α). Plates were run on an xMAP 200 Luminex (BioRad) and data was processed using BioPlex Manager (BioRad). Analyte concentrations were normalized according to tissue weight.

### ELISA analysis of placental lysates

Clarified placental lysates were prepared as described above, diluted 1:5, and assessed via MPO ELISA performed according to manufacturer’s instructions (SimpleStep, Abcam). MMP-1 and MMP-9 ELISAs were also performed according to vendor guidelines (Invitrogen) using 1:2 dilutions of clarified lysate. Plates were run on a SpectraMax i3x plate reader (Molecular Devices).

### Tandem mass tag (TMT) proteomics

Proteomic LC-MS/MS was performed in biological triplicate by the Fred Hutchinson Cancer Center's Proteomics and Metabolomics Core (see Supplemental Methods in S1 Text). In brief, 100 μg of protein from cytosolic, membrane, and secreted fractions were digested into tryptic peptides and labeled with TMT10plex isobaric tag reagents (Pierce). Peptides were generated from triplicate fractions of COH1 WT, COH1Δ*htrA*, and COH1Δ*htrA*/pDC123*htrA* with each receiving a unique isobaric tag allowing for peptide pooling by protein fraction. The tenth isobaric tag was used to label a standard comprised of an equal mix of peptides from all samples, allowing for normalization across all 27 samples. Pools were basic reverse phase (bRP) fractionated and analyzed by liquid chromatography/electrospray ionization-mass spectrometry (LC/ESI-MS/MS). MS data acquisition utilized synchronous precursor scanning along with real time searching via a *Streptococcus agalactiae* database (HG939456_1_StrepAgalactiae_COH1_NCBI_102521.fasta) using COMET Raw MS data were processed with Proteome Discoverer 2.5 (Thermo Scientific) and protein database searching was performed using a *Streptococcus agalactiae* protein database (HG939456_1_StrepAgalactiae_COH1_NCBI_102521.fasta) combined with a secondary database which included common contaminants (cRAPome). Within each protein fraction, changes in abundance between strains were assessed for significance using fold-change and *p-*value cutoffs of 1.5 and 0.05, respectively.

Kyoto Encyclopedia for Genes and Genomes (KEGG) functional annotations were assigned to *Streptococcus agalactiae* COH1 genome (GBCO_p1, NCBI) using FACoP.v2 (Functional Annotation and Classification of Proteins of Prokaryotes). An additional virulence category was produced using PubMed database searches for all significant hits.

### Purification of recombinant HtrA

A C-terminal His_6_-tagged HtrA variant was constructed using the expression vector pET32CK as follows. Primers used for PCR amplification are noted in parentheses throughout with the respective sequences listed in S1 Table. NcoI and NotI linearized pET32CK and *htrA* amplified from COH1 genomic DNA (*htrA* prot F/R) were combined via Gibson assembly (cloning kit, New England Biolabs) to generate pET32CK-*htrA*-H6. An HtrA^S237A^ catalytic mutant was achieved by replacing Ser237 with Ala by site directed mutagenesis using the QuikChange II kit (Agilent) (SDM*htrA*^S237A^ F/R), producing pET32CK-*htrA*^S237A^-H6. The resulting vectors were validated by sequencing (Plasmidsaurus) and transformed directly into BL21(DE3) gold cells (Agilent).

For purification of His_6_-tagged HtrA^S237A^ (HtrA^S237A^-H6), *E. coli* was grown at 37°C in LB supplemented with ampicillin. At OD_600_ of 0.6, cells were induced with 1 mM isopropyl-β-D-thiogalactopyranoside (IPTG) for 2 hours at 37°C. Cells were harvested by centrifugation and resuspended in urea solubilizing buffer (8 M urea, 0.1 M sodium phosphate (pH 8), 500 mM sodium chloride, 30 mM imidazol) and solubilized overnight at 4°C. Lysates were clarified by centrifugation at 23,000 x *g* for 30 minutes at 4°C and HtrA^S237A^-H6 was purified under denaturing conditions by affinity chromatography using Ni-NTA resin (Qiagen). The resin was washed in urea solubilizing buffer containing decreasing amounts of urea (8–0 M) for protein refolding. Following elution in 0.5 M imidazole, HtrA^S237A^-H6 was reconstituted in 50 mM sodium phosphate buffer (pH 8) and stored at -80°C until use. HtrA^S237A^-H6 was used for production of HtrA-specific polyclonal rabbit antiserum by Lampire Biologicals.

Due to rapid self-cleavage of HtrA-H6 (S3A Fig), it was necessary to employ an alternative purification strategy to maximize recovery of full-length protein. In brief, *E. coli* was induced by 0.1 mM IPTG at an OD_600_ of 0.6 for 1.5 hours at 37°Cand lysed in BugBuster protein extraction reagent (Millipore) according to manufacturer instructions. HtrA-H6 protein was purified under native conditions by affinity chromatography using Ni-NTA resin at 4°C to minimize protein cleavage. HtrA-H6 was washed in buffer (0.1 M sodium phosphate (pH 8), 500 mM sodium chloride, 30 mM imidazole), eluted as described above, and stored at -80°C until use.

### HtrA quantification

Rapid self-cleavage of HtrA-H6 during purification typically yielded low levels of full-length HtrA. Consequently, quantitative Western blot analysis was used to quantify HtrA-H6 for downstream enzymatic analysis. In brief, 20 ng HtrA^S237A^-H6 was separated alongside 1 μL of purified HtrA-H6 by SDS-PAGE and transferred to PVDF membranes for Western blot as described above. Membranes were subsequently probed with rabbit polyclonal HtrA antiserum and comparative densitometry analysis was performed using Fiji imaging analysis software to quantify HtrA-H6 based on signal intensity of HtrA^S237A^-H6 protein standard.

### Protease cleavage assays

To assess HtrA protease activity, β-casein, recombinant human fibronectin (Millipore Sigma), and recombinant Sip (MyBioSource) were used as substrates in protease cleavage assays. 10 μg of substrate was mixed with 10 ng of HtrA-H6 or HtrA^S237A^-H6 in 50 mM tris-HCl (pH 7.5, Sigma-Aldrich) overnight at 37°C. Reactions were inactivated by boiling in 1x LDS sample buffer (Invitrogen). 10 μL of each reaction was separated by SDS-PAGE (12% bis-tris with MES-SDS buffer, Invitrogen) and the banding pattern of the digest was analyzed via overnight staining with PageBlue protein staining solution (Thermo Scientific). Molecular weights of digested protein products were determined by comparison to PAGERuler pre-stained protein ladder (ThermoFisher). The assay was repeated in biological triplicate and a representative image is shown.

### HtrA toxicity to human vaginal epithelial cells

Human vaginal epithelial cells (hVECs) were maintained in keratinocyte serum-free medium (KSFM) supplemented with bovine pituitary extract and human recombinant EGF per vendor recommendations (aInvitrogen) with 1x Pen/Strep (Corning). Monolayers seeded in 6-well plates were treated with 10 ng vehicle, HtrA-H6, or HtrA^S237A^-H6 and incubated overnight at 37°C. Cell morphology was assessed via light microscope. The assay was repeated three times and representative images are shown.

### Statistical analysis

Statistical tests used are noted in the corresponding figure legend and were performed using GraphPad Prism, version 10.1.0 with a significance cutoff of *p* < 0.05.

Quantitative proteomic results were generated and normalized by Proteome Discoverer 2.5 (Thermo Scientific). To eliminate missing values, each reported quantitative intensity was adjusted by adding 1. Pairwise comparisons were assessed by *t*-test to calculate *p-*values and protein abundances changing by ≥ 1.5 that had a *p-*value ≤ 0.05 were considered significant.

## Supporting information

S1 Fig Deletion of *htrA* does not impact GBS growth *in vitro.* (A) A growth curve was generated via viable counts of WT COH1 and COH1Δ*htrA* GBS grown in TSB. Means of three biological replicates ± SEM were assessed for significance using two-way ANOVA with Šídác’s multiple comparisons test: ns *P* > 0.05. (B) WT and COH1Δ*htrA* stationary phase cultures were Gram stained and imaged at 100x. Scale bar indicates 10 μm and inset shows representative chained cocci.
(TIF)

S2 Fig GBS protein abundances in the cytosol and overlap of overall proteomic changes. (A) Volcano plots showing pairwise GBS strain comparisons analyzed for the cytosol fraction. Characters above each graph indicate relative HtrA levels of each strain. Proteins with significant changes in abundance (fold-change (FC) ≥ 1.5 & *p-*value < 0.05) are shown in dark purple and proteins meeting only one significance criterion are lilac. Bolded proteins have been previously linked to bacterial virulence and/or stress responses. (B-C) Tables indicate total number of proteins identified by LC-MS/MS, number of significant changes in abundance, and number of unique proteins undergoing changes of abundance within (B) each protein fraction or (C) each strain comparison. Venn diagrams summarize overlap of significant changes.
(TIF)

S3 Fig Purification of proteolytically active recombinant HtrA proteins and assessment in protease activity assays. (A) Recombinant HtrA-H6 or catalytically inactive HtrA^S237A^-H6 were purified from *E. coli* BL21(DE3) gold via Ni-NTA resin. Eluted fractions were assessed via Western blot using rabbit serum raised against recombinant HtrA^S237A^-H6 protein. Panel shows representative blot with catalytically active HtrA-H6 undergoing cleavage during purification. (B) Protease activity of HtrA-H6 and HtrA^S237A^-H6 was assessed using the model protease substrate β-casein (1:1000, HtrA:β-casein) compared to single protein controls. The assay was repeated three separate times and a representative Coomassie stained gel is shown. (C) Densitometry of full-length β-casein was performed in Fiji. Data shows means ± SEM of three replicates, with significance determined by ordinary one-way ANOVA with Tukey’s multiple comparison test: * *P* < 0.05. Trending (*P* < 0.1) statistics are shown.
(TIF)

S1 Table Primers used for strain manipulation. (DOCX)

S1 Text Supplemental Methods. (DOCX)

S1 Data Proteomics Data. (XLSX)

S2 Data Significant Proteins and KEGG Functions. (XLSX)
