# Supplementary figures and images for: The serine protease HtrA regulates Group B Streptococcus virulence and affects the host response to infection
Source: PLoS Pathog. 2025 Oct 6;21(10):e1013562. doi: 10.1371/journal.ppat.1013562 (PMC12520345; doi:10.1371/journal.ppat.1013562)

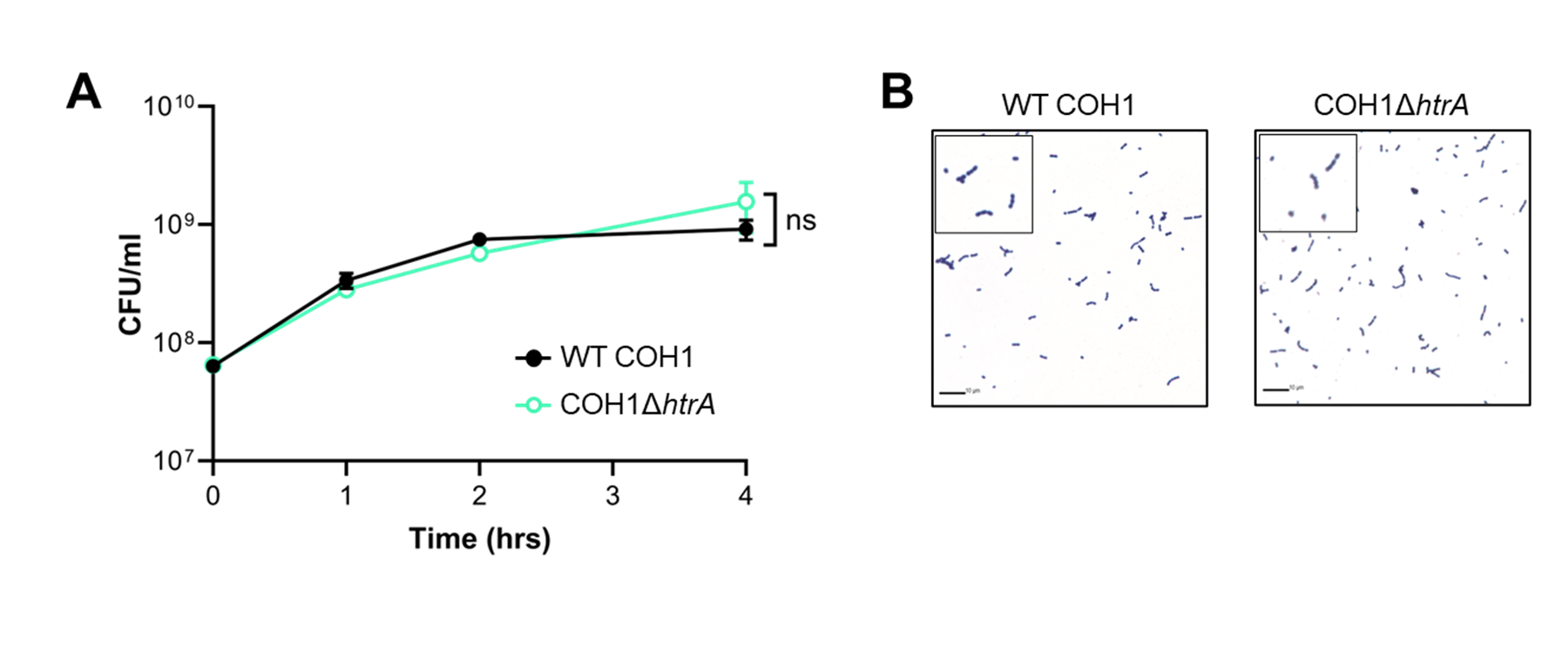

Supplement: S1 Fig — (A) A growth curve was generated via viable counts of WT COH1 and COH1ΔhtrA GBS grown in TSB. Means of three biological replicates ± SEM were assessed for significance using two-way ANOVA with Šídác’s multiple comparisons test: ns P > 0.05. (B) WT and COH1ΔhtrA stationary phase cultures were Gram stained and imaged at 100x. Scale bar indicates 10 μm and inset shows representative chained cocci. (TIF) [file ppat.1013562.s001.tif]

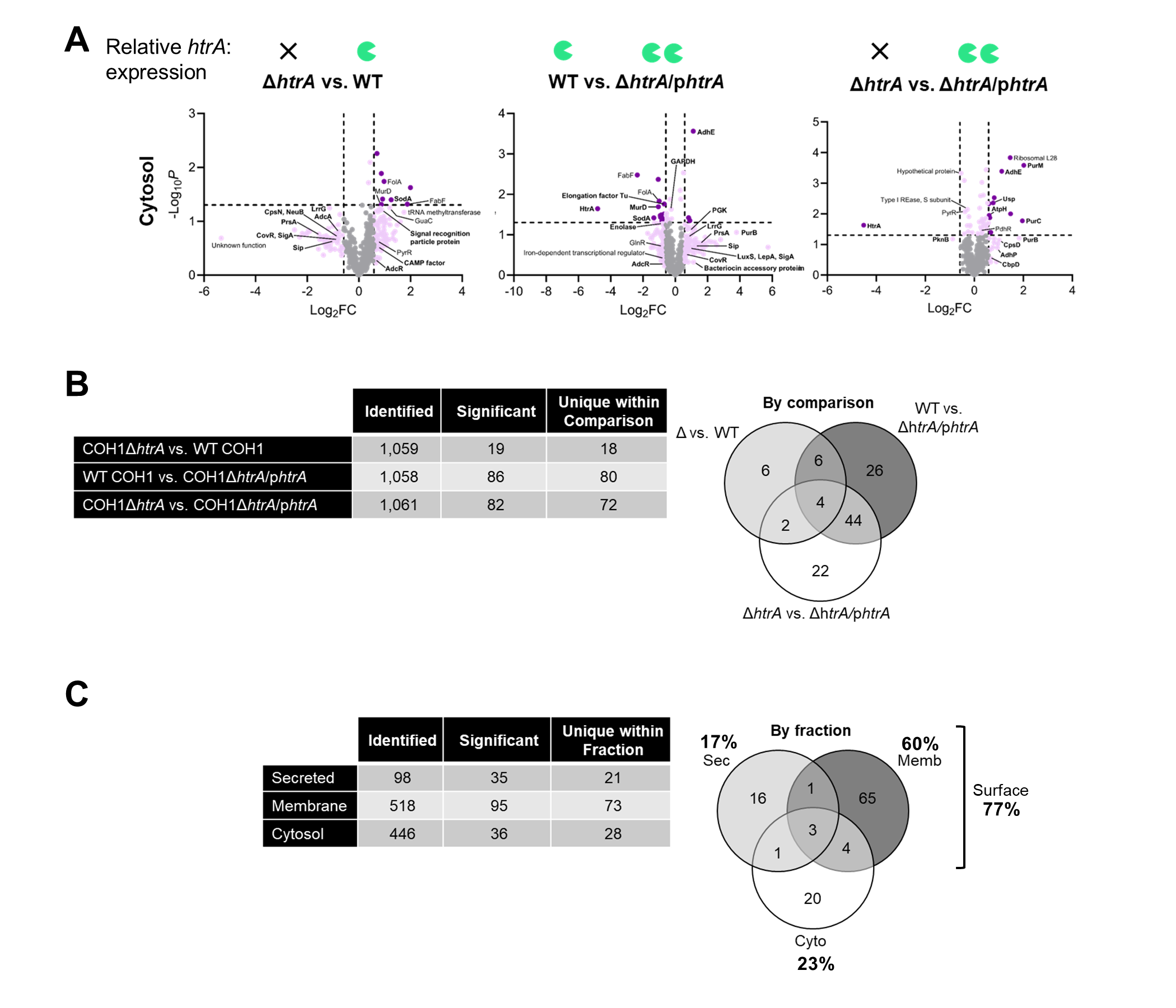

Supplement: S2 Fig — (A) Volcano plots showing pairwise GBS strain comparisons analyzed for the cytosol fraction. Characters above each graph indicate relative HtrA levels of each strain. Proteins with significant changes in abundance (fold-change (FC) ≥ 1.5 & p-value < 0.05) are shown in dark purple and proteins meeting only one significance criterion are lilac. Bolded proteins have been previously linked to bacterial virulence and/or stress responses. (B-C) Tables indicate total number of proteins identified by LC-MS/MS, number of significant changes in abundance, and number of unique proteins undergoing changes of abundance within (B) each protein fraction or (C) each strain comparison. Venn diagrams summarize overlap of significant changes. (TIF) [file ppat.1013562.s002.tif]

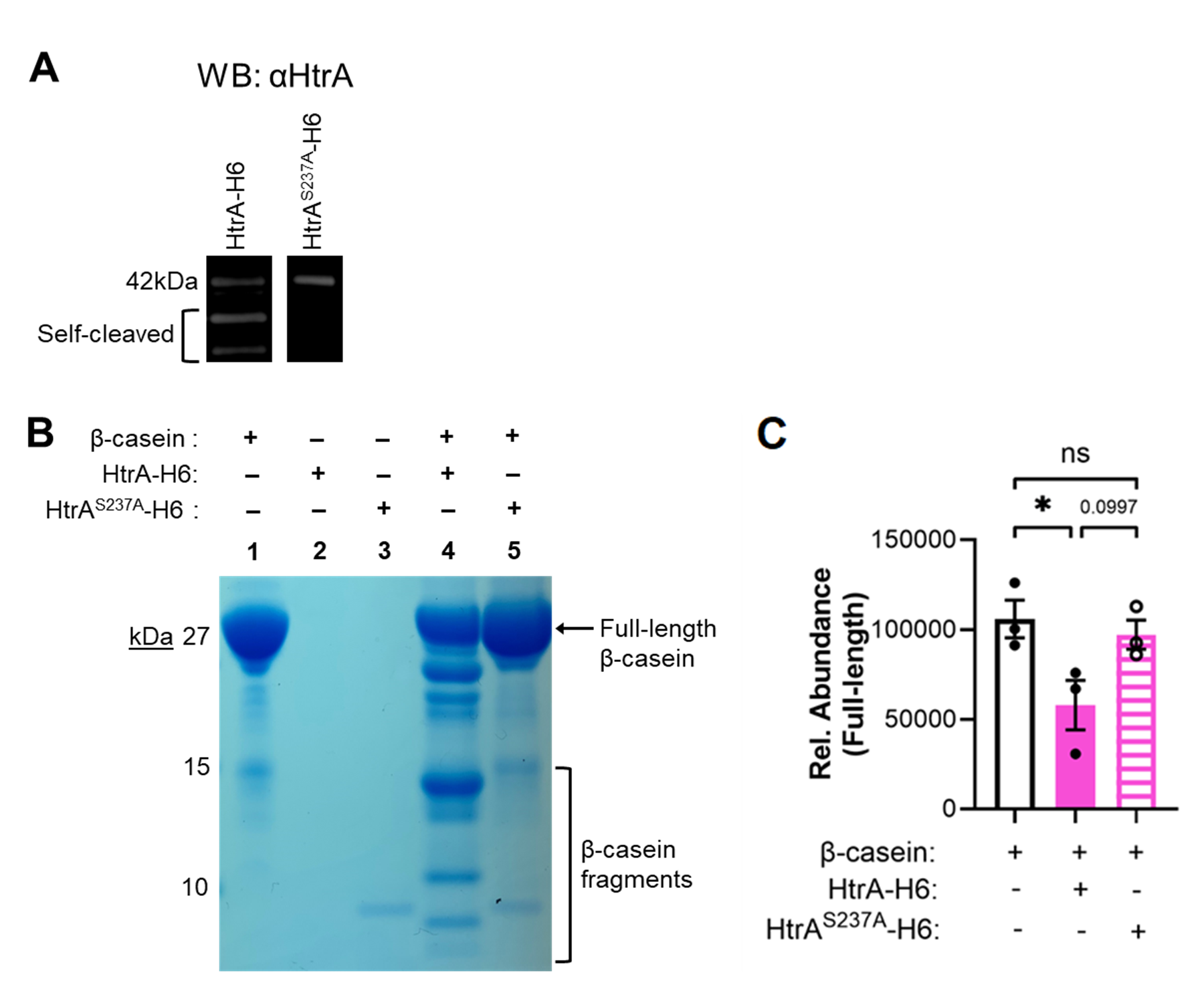

Supplement: S3 Fig — (A) Recombinant HtrA-H6 or catalytically inactive HtrAS237A-H6 were purified from E. coli BL21(DE3) gold via Ni-NTA resin. Eluted fractions were assessed via Western blot using rabbit serum raised against recombinant HtrAS237A-H6 protein. Panel shows representative blot with catalytically active HtrA-H6 undergoing cleavage during purification. (B) Protease activity of HtrA-H6 and HtrAS237A-H6 was assessed using the model protease substrate β-casein (1:1000, HtrA:β-casein) compared to single protein controls. The assay was repeated three separate times and a representative Coomassie stained gel is shown. (C) Densitometry of full-length β-casein was performed in Fiji. Data shows means ± SEM of three replicates, with significance determined by ordinary one-way ANOVA with Tukey’s multiple comparison test: * P < 0.05. Trending (P < 0.1) statistics are shown. (TIF) [file ppat.1013562.s003.tif]
