# Supplementary material for: The serine protease HtrA regulates Group B Streptococcus virulence and affects the host response to infection
Source: PLoS Pathog. 2025 Oct 6;21(10):e1013562. doi: 10.1371/journal.ppat.1013562 (PMC12520345; doi:10.1371/journal.ppat.1013562)
Supplement: S1 Table — (DOCX) [file ppat.1013562.s004.docx]

**S1 Table. Primers used for strain manipulation.**

| **Primer name** | **Primer sequence** |
| --- | --- |
| *htrA* upstream forward | GAA TTC CTG CAG CCC AGT ACT CTT AAT ATT ATA TCA GAA AAA AAA G |
| *htrA* upstream reverse | TTT AGC CAT AAA TAG CTA CCT CCG TAA ATT TTT ATA C |
| *htrA* downstream forward | ATT GTT TTA GTC AAT TCT AGT TAT TTT AAC TAA GAT ATA GTA AAA TTA AA |
| *htrA* downstream reverse | ACT AGT GGA TCC CCC TAA AGT TTT CCA CAG ACT GTG |
| *kanR* forward | GTA GCT ATT TAT GGC TAA AAT GAG AAT ATC AC |
| *kanR* reverse | CTA GAA TTG ACT AAA ACA ARR CAT CCA GTA AAA TAT AAT ATT TTA TTT TC |
| pHY304 forward | GGG GGA TCC ACT AGT TCT AG |
| pHY304 reverse | GGG CTG CAG GAA TTC GAT |
| *htrA* comp forward | GAA AAA CAA ACA GAG GTT TTA CGG AGG TAG CTA TTT ATG GTG AAA A |
| *htrA* comp reverse | CGG GCC CGG GTA CCG CAT GCT TAG TTA TTT GCT CGT TGT TTA G |
| pDC123 forward | AAC CTC TGT TTG TTT TTC GCC GC |
| pDC123 reverse | GCA TGC GGT ACC CGG GC |
| pBluescript forward | AAA AAG GGG AAT AGT TAT GAG TAT CTC TAG AAC CGC GGT GGA GCT CCA G |
| pBluescript reverse | CGG GAA CCC GCA GAT AAA ACC ATA CGG ATC CGG CCC GGT ACC CAA TTC G |
| *htrA* prot forward | ATA CCC ATG GCT TTT GTC ATG AAT CAT AAT GAC AAT ATT C |
| *htrA* prot reverse | AGT GCG GCC GCG TTA TTT GCT C |
| SDM*htrA*S237A forward | GTG CTC CAC CTG CAT TAC CAG GAT TGA TAG CAG CA |
| SDM*htrA*S237A forward | TGC TGC TAT CAA TCC TGG TAA TGC AGG TGG AGC AC |
| All primers were purchased from IDT. | |
