# Supplementary material for: The serine protease HtrA regulates Group B Streptococcus virulence and affects the host response to infection
Source: PLoS Pathog. 2025 Oct 6;21(10):e1013562. doi: 10.1371/journal.ppat.1013562 (PMC12520345; doi:10.1371/journal.ppat.1013562)
Supplement: S1 Text — (DOCX) [file ppat.1013562.s005.docx]

**S1 Text: Supplemental Methods**

*Sample preparation for TMT proteomic analysis*

100 μg of GBS protein fractions (1 μg/μL) in 100 mM ammonium bicarbonate were reduced by tris (2-carboxyethyl) phosphine (final concentration of 5 mM) for 15 minutes at room temperature, then alkylated by 2-chloroacetamide (final concentration of 10 mM) in the dark for 30 minutes at room temperature. Excess 2-chloroacetamide was quenched by dithiothreitol (10 mM final concentration) at room temperature for 30 minutes. Proteins were digested by 1 μg rLys-C (Promega) for 4 hours at 37 ℃, followed by 1 μg of trypsin (Promega) at 37 ℃ overnight. The peptides were desalted over Oasis HLB C18 1 cc cartridges (Waters) and dried in a SpeedVac.

Next, the peptides were TMT-labelled to allow multiplexing. To this end, dried peptide samples were first resuspended in 70 μL of 50 mM HEPES (pH 8.5), and 800 μg TMT10plex isobaric tag labeling reagents (Pierce) were resuspended in 30 μL acetonitrile and added to the digested peptide solution [30% (v/v) final organic concentration]. Each biological replicate for each GBS strain was given a unique isobaric tag to allow pooled fractions to be analyzed in 3 runs (9 experimental samples per run). A standard comprised of equal amounts of each sample’s un-tagged peptide mixture was labeled with the final isobaric tag to allow normalization across each run. The peptide mixtures were incubated with their isobaric tag for 1 hour at room temperature. To check labeling efficiency, 2 μg from each sample was combined, dried, run over a C18 ZipTip (Millipore), and analyzed via LC/MS to determine equalization amounts. The sufficiently labeled reactions were quenched with 0.3% (v/v) hydroxylamine for 15 minutes, then the TMT-labeled samples were pooled by fraction (1:1 ratio based on equalization amounts), and concentrated on a SpeedVac to remove acetonitrile. A 20% fraction of the dried pool was desalted over an Oasis HLB C18 1cc cartridge (Waters) and dried with a SpeedVac.

For basic reverse phase (bRP) fractionation, the 20% de-salted TMT-labeled pool was resuspended in 100 µl 10 mM ammonium bicarbonate (pH 8) and acetonitrile (95:5, v/v), and loaded onto a Zorbax Extend-C18 column (2.1 mm x 150 mm, 5 µm; Agilent) connected to a Vanquish Horizon UHPLC (ThermoFisher). The sample was gradient-eluted at a flow-rate 250 µl/min over 55 minutes with a combination of 10 mM ammonium carbonate (solvent A) and acetonitrile (B) using the following gradient: 0 to 5 minutes B was held at 1%, 5 to 55 minutes B varied from 5% to 40%, 55-60 minutes B gradually increased to 90% B, then held at 90% B from 60-65 minutes. The UV signal was monitored at 210 nm. Fractions were collected every 50 seconds resulting in a total of 96 fractions, which were pooled by combining every eighth fraction (pool 1 combines fractions 1, 9, 17, 25, 33, 41, 49, 57, 65, 73, 81, and 89; pool 2 combines fractions 2, 10, 18, 26, 34, 42, 50, 57, 66, 74, 82, and 90; etc.). The final pools were vacuum centrifuged to near-dryness and resuspended in 45 μl of 2% acetonitrile in 0.1% formic acid for LC-MS analysis.

*Liquid chromatography-mass spectrometry*

1.5 μL of each TMT-labeled concatenated pool were loaded for liquid chromatography/electrospray ionization-mass spectrometry (LC/ESI-MS/MS) using an Easy1200 nLC system (Thermo Scientific) coupled to a Orbitrap Eclipse Tribrid mass spectrometer with FAIMS Pro interface (Thermo Scientific). In-line de-salting was accomplished using a reversed-phase trap column (100 μm x 20 mm) packed with Magic C_18_AQ resin (5 μm, 200 Å; Michrom Bioresources) and peptides were separated by a reversed-phase column (75 μm x 270 mm) packed with ReproSil-Pur C_18_AQ (3-μm, 120 Å resin; Dr. Maisch HPLC) directly mounted on the electrospray ion source. A 180-minute gradient from 4% to 44% B at a flow rate of 300 nl/minute was used for chromatographic separations. A spray voltage of 2300 V was applied to the electrospray tip in-line with a FAIMS Pro source using varied compensation voltage (-40 V, -60 V, -80 V) while the instrument was operated in data-dependent mode. MS survey scans were performed on the Orbitrap (normalized AGC target value 300%, resolution 120,000, max injection time auto) with a 3 second cycle time. MS/MS spectra were detected in the linear ion trap (normalized AGC target value 100%, max injection time 50 ms) by collision-induced dissociation (CID) activation with a normalized collision energy (NCE) of 35% using turbo speed scan. Selected ions were dynamically excluded for 60 seconds after a repeat count of 1.

Following MS2 acquisition, spectra were searched in real time against a *Streptococcus agalactiae* COH1 protein database (HG939456_1_StrepAgalactiae_COH1_NCBI_102521.fasta) using COMET (1). Searches were performed for trypsin specificity with maximum missed cleavages set to 1. Oxidation (+15.9949 Da on M) was set as a dynamic modification. Static modifications included TMT (+229.1629 Da on K) and carbamidomethyl (+57.0215 Da on C). Maximum search time was 35 ms. Scoring thresholds were set to the following: Xcorr 1.4, dCn 0.1, precursor PPM 10, and charge state 2. Synchronous precursor selection (SPS)-MS3 was collected on the top 10 most intense ions detected in the MS2 spectrum. SPS-MS3 precursors were subjected to higher energy collision-induced dissociation for fragmentation with NCE of 65% and analyzed using the Orbitrap (normalized AGC target value 400%, resolution 50,000, maximum injection time 86 ms).

*TMT proteomics data analysis*

Quantitative proteomic data analysis was performed using Proteome Discoverer 2.5 (Thermo Scientific) and searched against a *Streptococcus agalactiae* COH1 protein database (HG939456_1_StrepAgalactiae_COH1_NCBI_102521.fasta) appended to include common contaminants (Contaminant Repository for Affinity Purifiaction, cRAPome) (2). Searches were performed for trypsin specificity with maximum missed cleavages set to 2. Precursor ion tolerance and fragment ion tolerance were set to 10 ppm and 0.6 Da, respectively. Dynamic peptide modifications included oxidation (+15.995 Da on M) and dynamic modifications on the protein terminus included acetyl (+42.-11 Da on N-terminus), Met-loss (-131.040 Da on M), and Met-loss+Acetyl (-89.030 Da on M), plus static modifications TMT (+229.1629 Da on any N-terminus), TMT (+229.1629 Da on K), and carbamidomethyl (+57.0215 on C). Database searches were performed by SEQUEST HT (3) and results were run through Percolator for peptide validation and false discovery rate calculation (4).

Raw data were normalized by Proteome Discoverer and intensities were adjusted by adding a value of 1 to each intensity to avoid missing values. *P*-values for pairwise comparisons with adjusted intensity were calculated by t-test. Fold-change ≥ 1.5 and *P*-value ≤ 0.05 were used to identify significant changes in abundance. Data was log-transformed (Log_2_FC or -Log_10_*P*) to produce volcano plots in GraphPad Prism (version 10.1.0).

Kyoto Encyclopedia for Genes and Genomes (KEGG) functional annotations were assigned to the *Streptococcus agalactiae* COH1 genome (GBCO_p1) using FACoP.v2 (Functional Annotation and Classification of Proteins of Prokaryotes,). An additional virulence category was produced through to PubMed database searches.

**SUPPLEMENTAL REFERENCES**

1. Eng JK, Jahan TA, Hoopmann MR. Comet: an open-source MS/MS sequence database search tool. Proteomics. 2013;13(1):22-4.

2. Mellacheruvu D, Wright Z, Couzens AL, Lambert JP, St-Denis NA, Li T, et al. The CRAPome: a contaminant repository for affinity purification-mass spectrometry data. Nat Methods. 2013;10(8):730-6.

3. Eng JK, McCormack AL, Yates JR. An approach to correlate tandem mass spectral data of peptides with amino acid sequences in a protein database. J Am Soc Mass Spectrom. 1994;5(11):976-89.

4. Kall L, Canterbury JD, Weston J, Noble WS, MacCoss MJ. Semi-supervised learning for peptide identification from shotgun proteomics datasets. Nat Methods. 2007;4(11):923-5.
